# Supplementary material for: Forest management in southern China generates short term extensive carbon sequestration
Source: Nat Commun. 2020 Jan 8;11:129. doi: 10.1038/s41467-019-13798-8 (PMC6949300; doi:10.1038/s41467-019-13798-8)
Supplement: Supplementary file 1 — Supplementary Information [file 41467_2019_13798_MOESM1_ESM.pdf]

Supplementary Information

Forest management in southern China generates short-term extensive carbon sequestration

Tong et al.

includes:

Supplementary Figures 1-11

Supplementary Tables 1-3

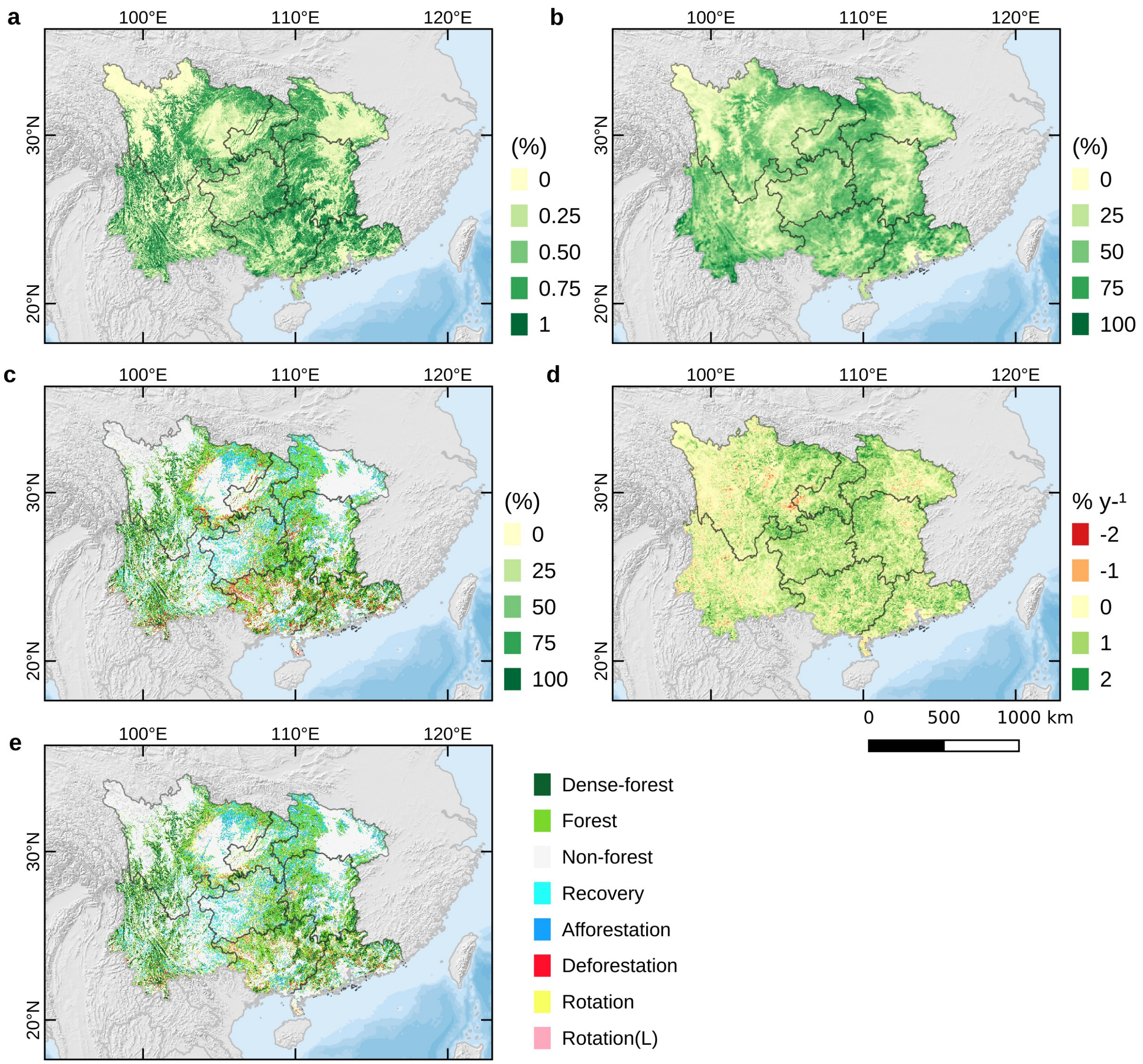

Supplementary Figure 1: Forest maps. a, Mean forest probability (2002–2017) at a resolution of 500 m. **b**, Mean tree cover (2002–2017) at a resolution of 0.05° from ref [1]. **c,** Tree cover (2010) at a resolution of 30 m from ref [2]. **d,** Tree-cover changes for 2002–2017 at a resolution of 0.05° from ref [1]. **e,** Types of forest management derived from (a).

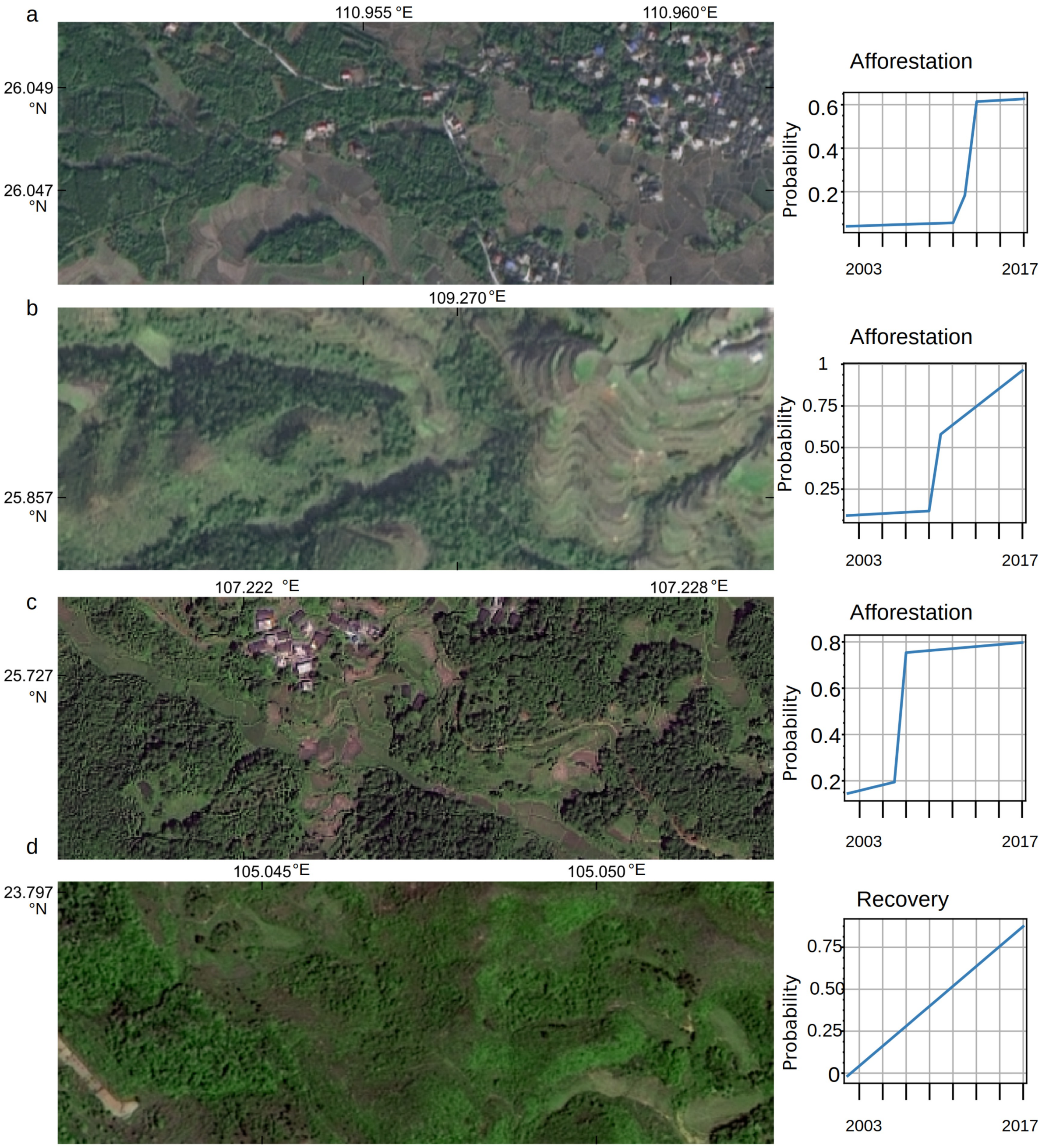

Supplementary Figure 2: *Afforestation* and *Recovery* in GF-1 images. Changes in forest probabilities from 2002 to 2017 are shown at the right hand side. Examples show (a-c) tree plantings around villages and on cropland, which are common in southern China. d, An area is typically natural forests if tree cover slowly increases.

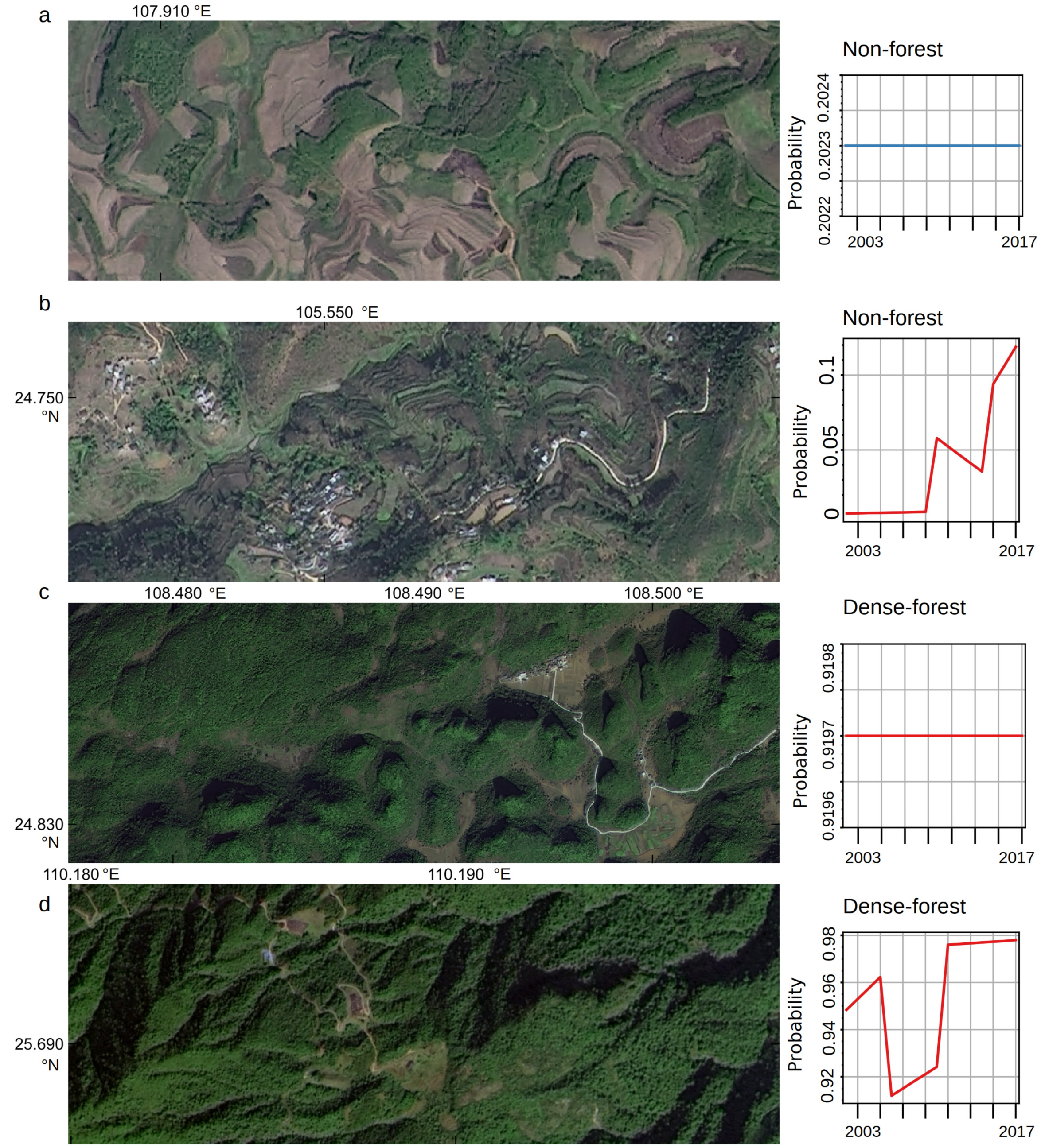

Supplementary Figure 3: Non-forest, forest and Dense forest in GF-1 images. Changes in forest probabilities from 2002 to 2017 are shown at the right hand site. a, *Non-forest*: some agricultural plantings e.g. sugarcane, are tall and have high greenness but are not similar to forest areas. b, *Non-forest*: tree plantings on farmland do not necessarily qualify as a forest if the increase in forest probability is too weak. c, A *Dense forest* in the karst area remains undisturbed during 2002–2017. d, This *Dense forest* is only weakly disturbed.

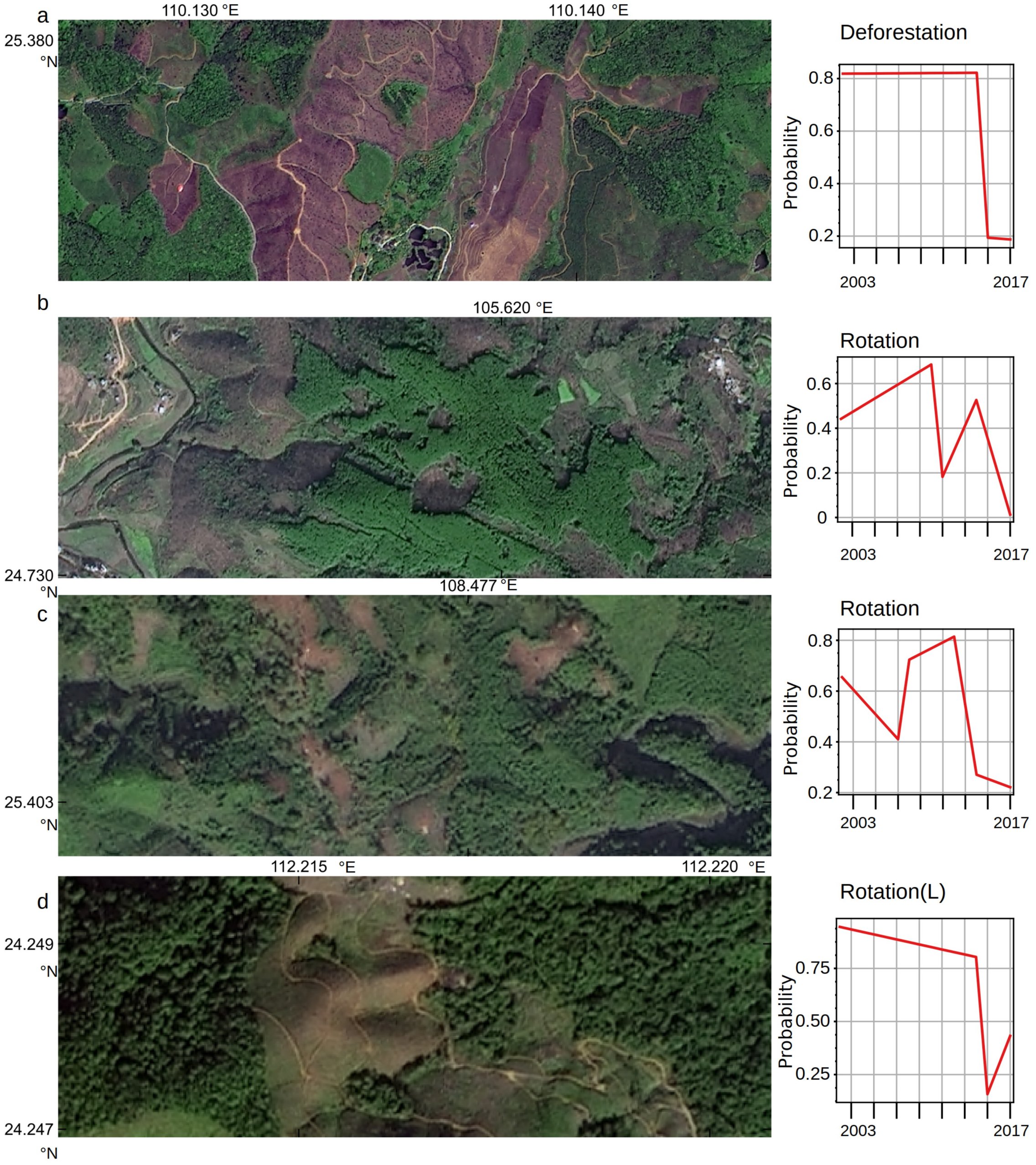

Supplementary Figure 4: Forestry actions in GF-1 images. Changes in forest probabilities from 2002 to 2017 are shown at the right hand site. a, *Deforestation* is mapped if cover greatly decreases with no increases. **b and c**, *Rotation* implies changes between forest (probability >50%) and non-forest (<50%). d, $Rotation_L$ implies a large-scale decrease ("clear-cut"), similar to deforestation. The difference to deforestation is that slopes of segments can also be positive.

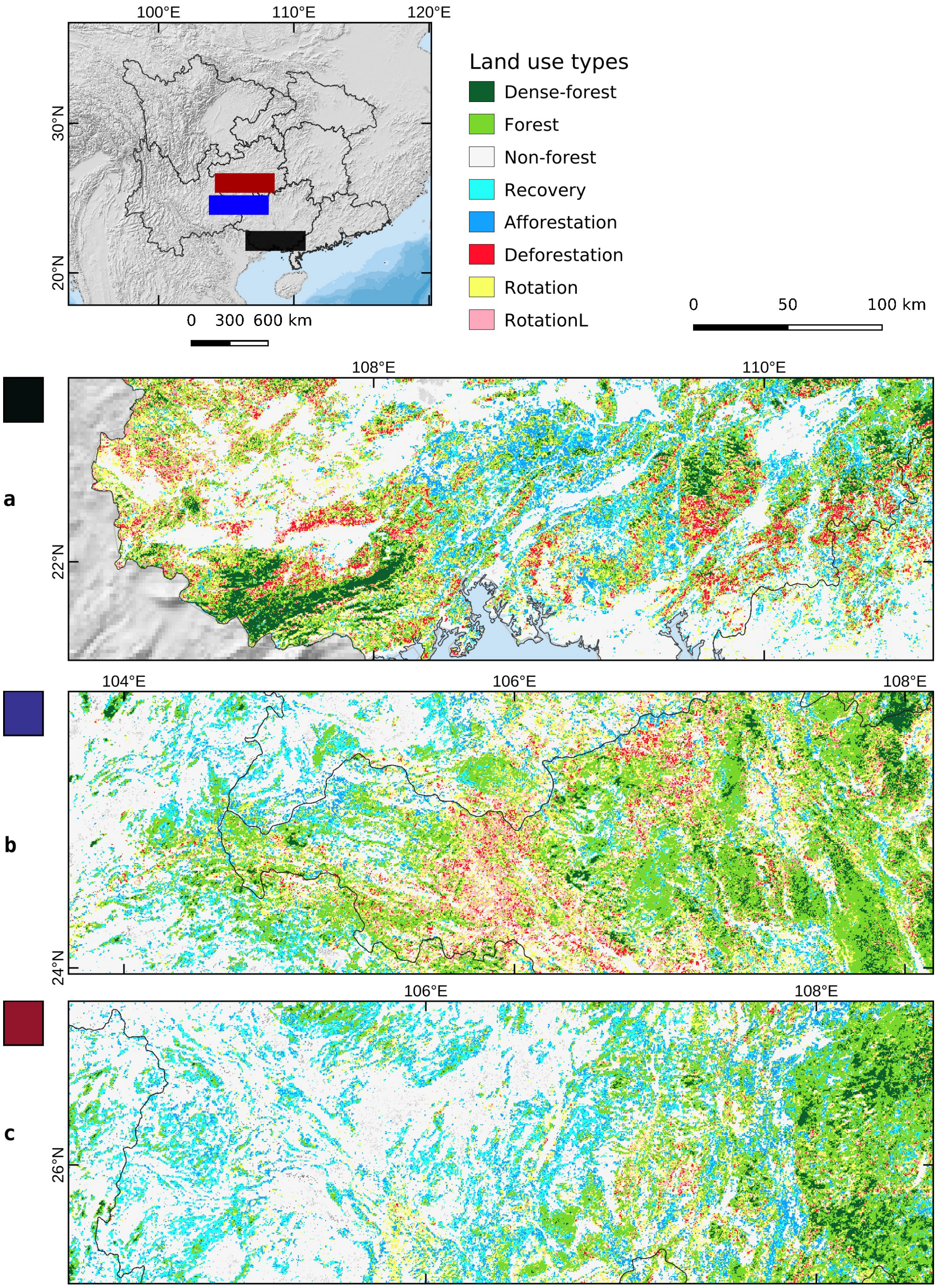

Supplementary Figure 5: Example areas showing forest types 1. a, The coastal area around Nanning (Guangxi) is a very dynamic are with large plantation areas. **b,** The northern part of Guangxi is characterised by large areas of forest removal, but also new plantations can be seen. **c,** The karst area in Guizhou is a hot spot of natural recovery areas.

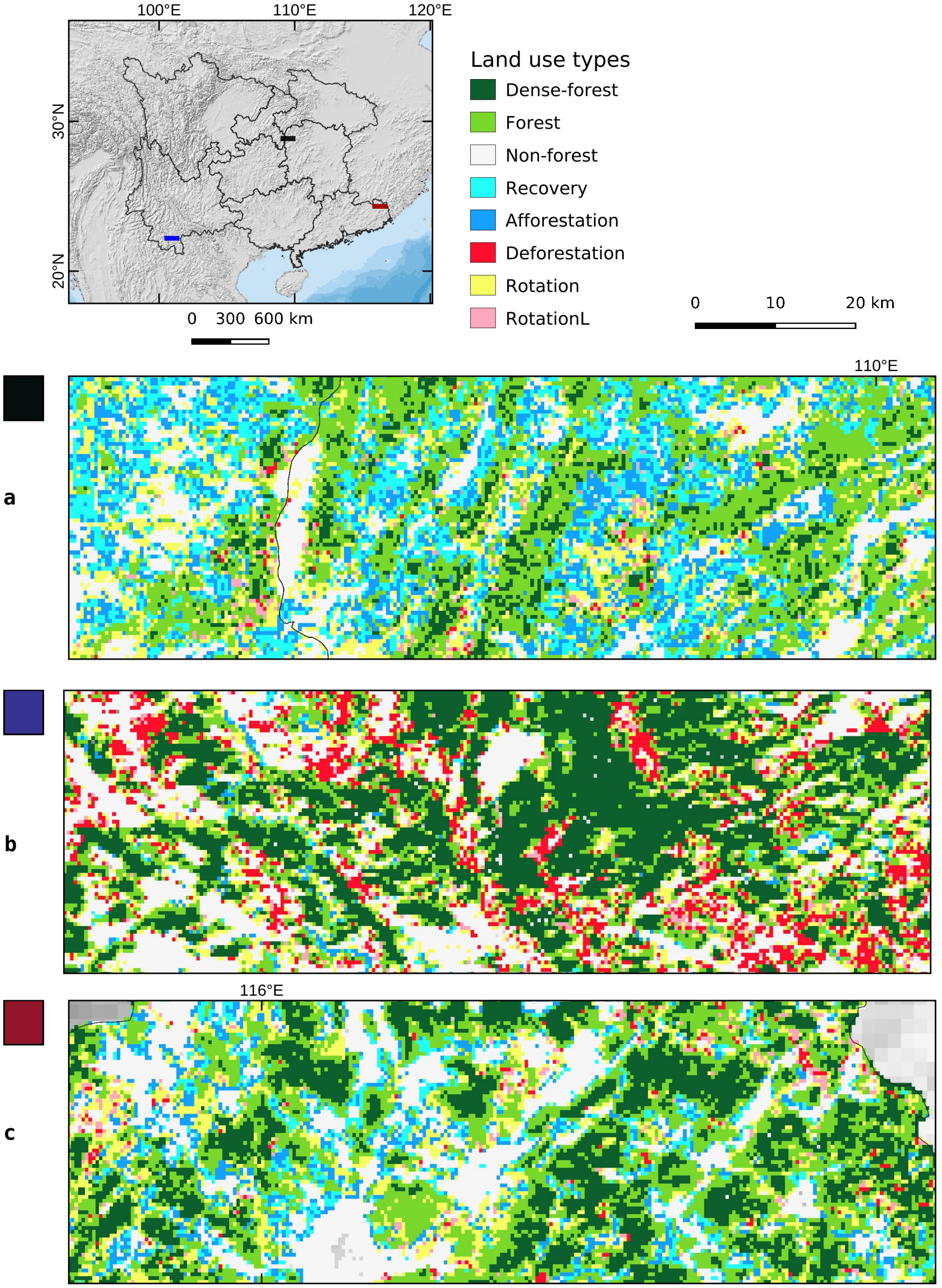

Supplementary Figure 6: Example areas showing forest types 2. a, New forests are often filling the gaps between existing forests. **b,** *Dense forests* in Yunnan are threatened by deforestation spreading from the valleys. **c**, Contrasting to the last example, forestation is spreading here from the settlements increasing the existing forest areas.

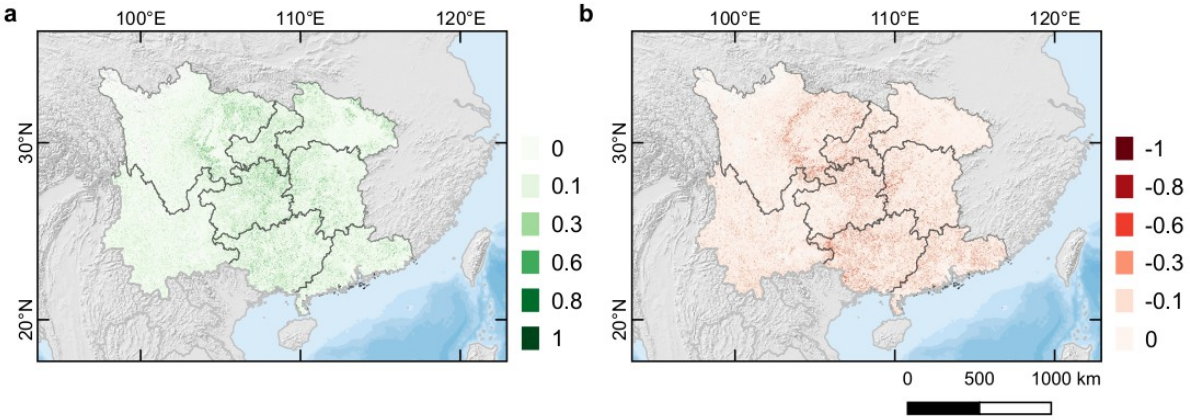

Supplementary Figure 7: Indices of managed forests. a, Map of *managed forest increase*, supposedly tree plantings. **b,** Map of *managed forest decrease*, supposedly tree harvests. The indices ranging from 0 to 1 (positive) and from 0 to -1 (negative) are calculated by dividing the strongest positive/negative segment (derived from the LandTrendr algorithm based on forest probability) by the duration of the segment (i.e. the period of years). A strong and rapid change in forest probability is most likely caused by human management, i.e. tree planting and harvesting.

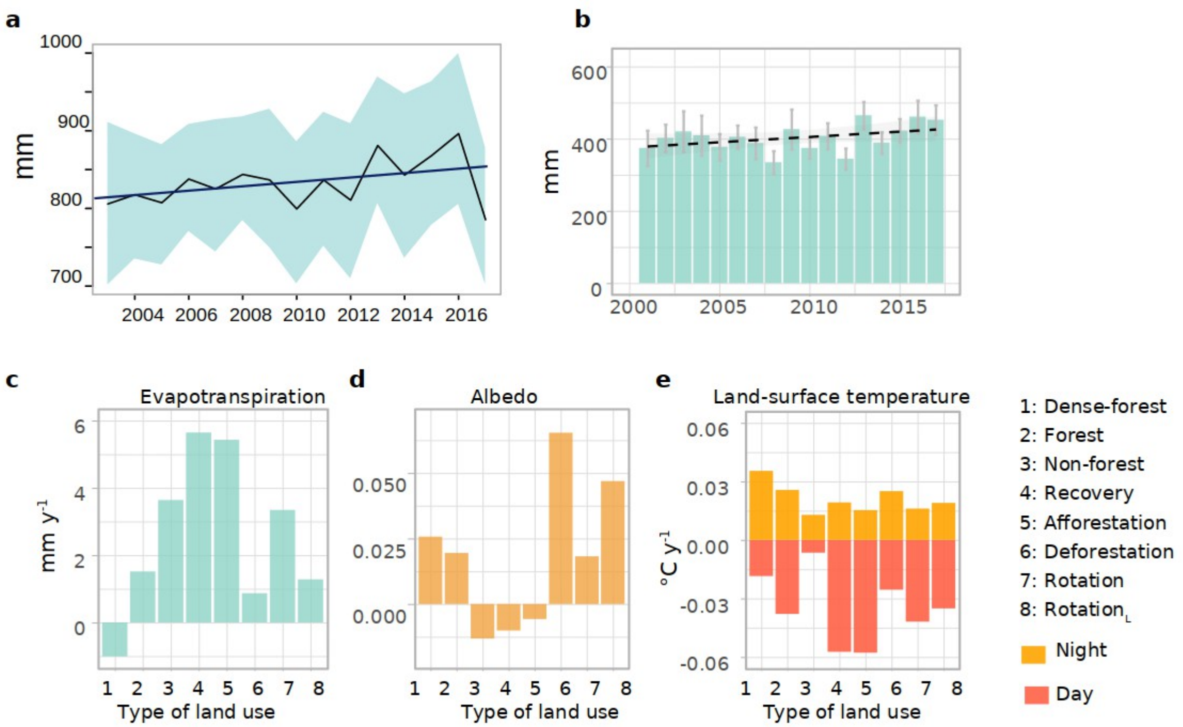

Supplementary Figure 8: a, Actual evapotranspiration (mean, 25% and 75% shown as shaded areas) for the study area from the GLEAM data set [3]. **b,** Actual evapotranspiration (error bars are standard deviations) from MOD16 c6. **c,** Actual evapotranspiration from MOD16 for different land use types. **d,** Albedo from MCD43 c6 (annual median of daily values). **e**, Land surface temperature from MOD11 c6 (annual median of daily values). Pixel number for eight provinces = 8573649.

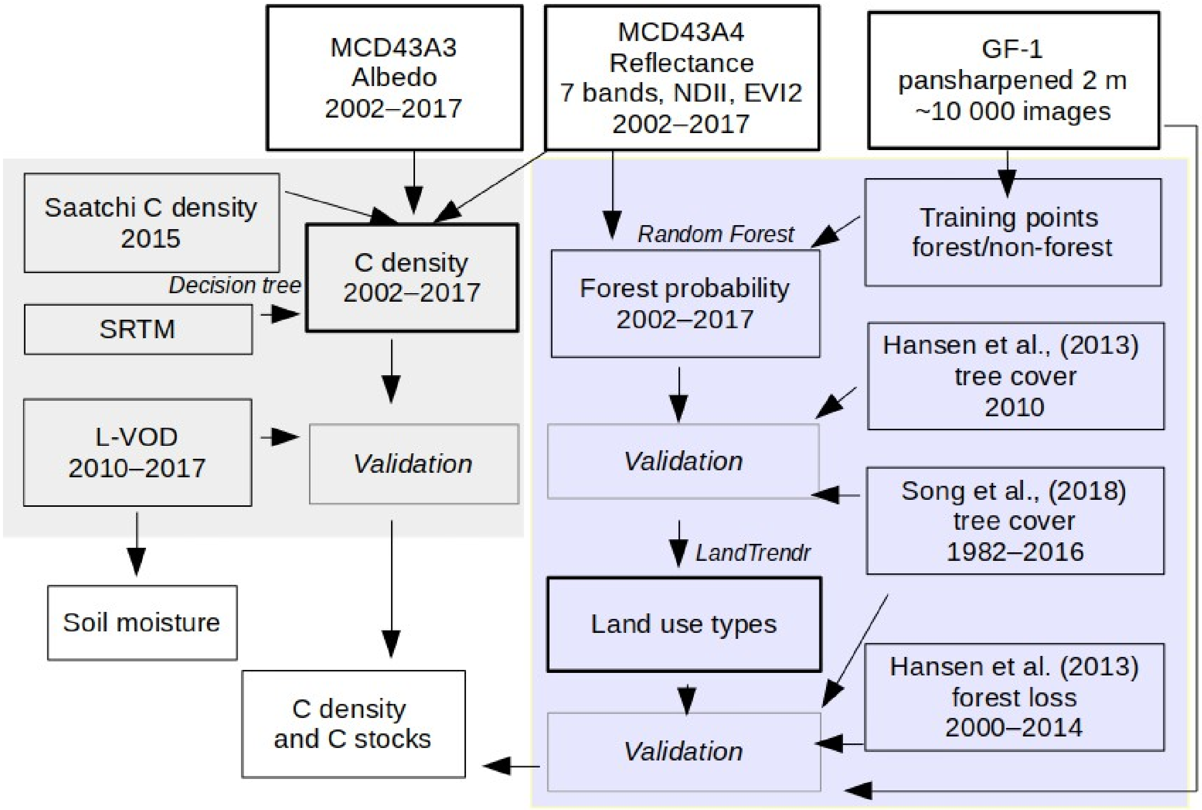

Supplementary Figure 9: Flowchart showing the work-flow and data sets used.

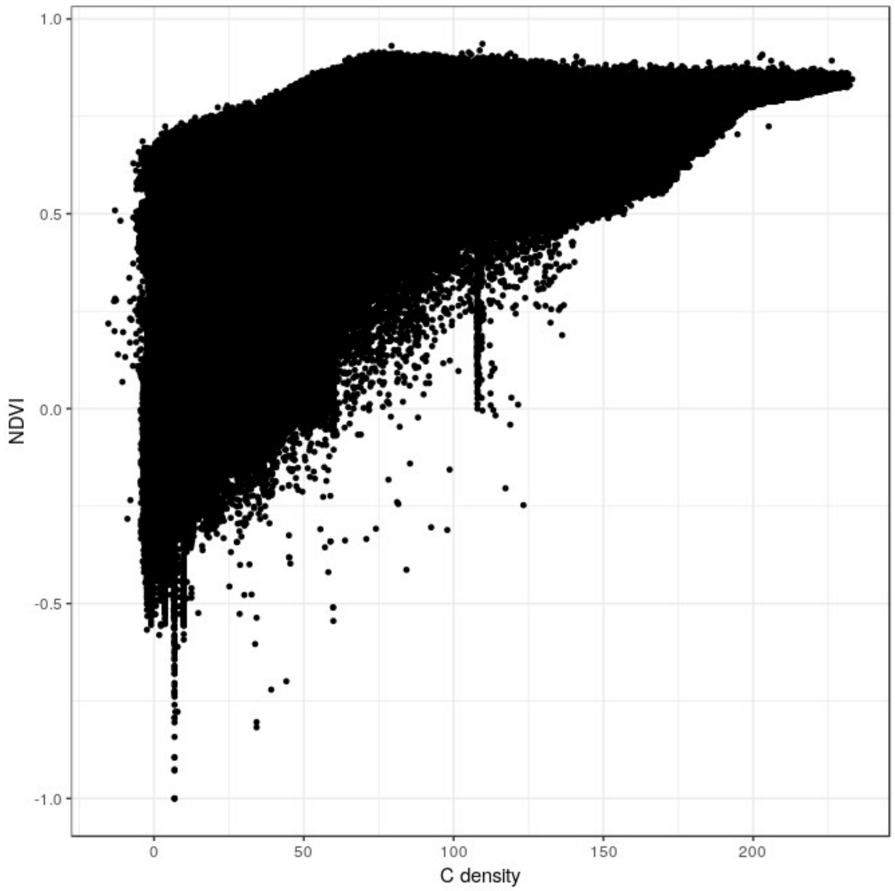

Supplementary Figure 10: Comparison between NDVI and C-density. While NDVI saturates in more densely vegetated areas, this is not the case for our C-density product, here shown for 2017 for the study area.

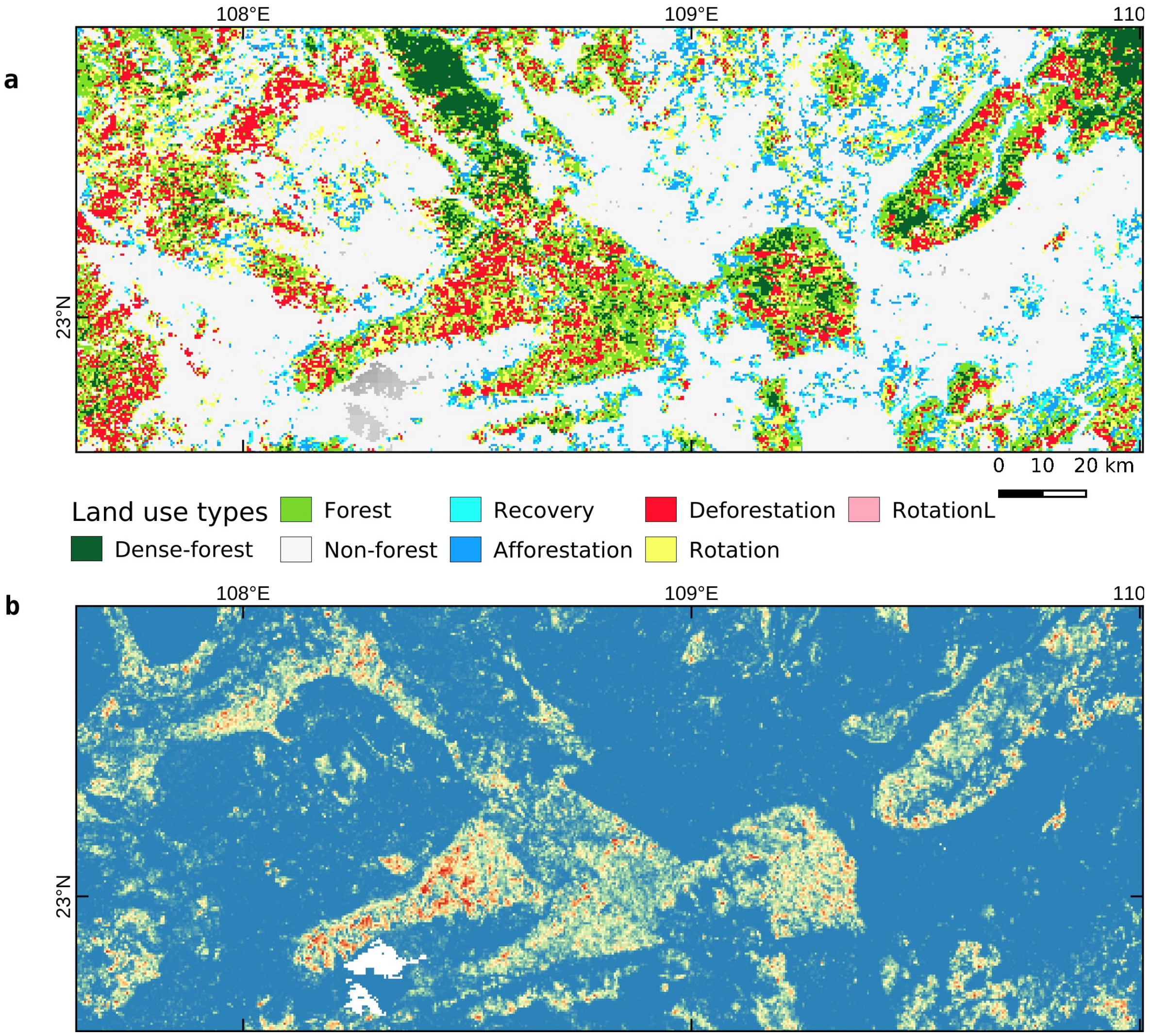

Supplementary Figure 11: Comparison of our forest type classification with the forest loss map by ref[2]. **a,** The forest type map from our study. The deforestation areas agree well with **b,** the forest loss map by ref [2]. The different reddish colours show different loss years.

Supplementary Table 1: Tree cover dynamics derived from VCF5KYR[1] for land use types. The units of the first two columns are changes in forest cover per year, and the numbers in brackets represent the bare-ground dynamics (percent bare-ground change per year) derived from the same product.

Type	Slope 1982–2014 (% cover y^{-1})	Slope 1999–2016	1982 (% cover)	1999 (% cover)	2016 (% cov-er)
All	+0.28 (-0.05)	+0.49 (-0.08)	21 (9)	27 (4)	38 (2)
Dense forest + Forest	+0.4 (-0.03)	+0.60 (-0.03)	40 (3)	44 (3)	58 (1)
Non-forest	+0.19 (-0.06)	+0.30 (-0.13)	12 (10)	14 (9)	21 (3)
Recovery + Afforestation	+0.45 (-0.08)	+0.75 (-0.13)	21 (9)	26 (5)	40 (1)

Supplementary Table 2: Mean (2002–2017) numbers for ET, albedo, LST, and C density from MODIS products. The percent cropland was derived from the ESA CCI land-cover map for 2000. The mean duration of segments shows the average (± S.D.) number of years without disturbance.

Type	ET	Albedo (%)	LST day/night (℃)	Area km²	Cropland (%)	Mean duration of segments
Dense forest	428	10.07	18.56/11.03	188 111	1	9.3±5
Forest	436	10.9	20.72/12.28	439 000	9	8.5±4.8
Non-forest	366	13.8	21.79/10	939 051	51	8.3±4.8
Recovery	418	11.8	22.82/13.24	116 946	37	12.5±3.8
Afforestation	420	12.1	23.2/13.61	158 198	45	5.2±1.2
Deforestation	498	12.6	23.78/15.63	34 309	16	10.4±4.3
Rotation	428	12.1	23.28/14.35	222 803	34	4.6±1.3
Rotation_L	461	12.4	24/15.65	39 760	25	4.7±1.3

Supplementary Table 3: Mean C densities from the MODIS maps and uncertainties for different land use types (RMSE for the 95% confidence interval).

Type	Estimated C density (Mg C ha^{-1})	Saatchi C density (Mg C ha^{-1})	Uncertainty (RMSE) (Mg C ha^{-1})
All	47	43	11
Dense forest	106	75	14
Forest	75	75	14
Non-forest	22	21	8
Recovery	47	46	12
Afforestation	44	45	12
Deforestation	62	62	13
Rotation	52	51	13

1. Song, X.-P. *et al.* Global land change from 1982 to 2016. *Nature* **560**, 639–643 (2018).
2. Martens, B. *et al.* GLEAM v3: satellite-based land evaporation and root-zone soil moisture. *Geoscientific Model Development* **10**, 1903–1925 (2017).
3. Hansen, M. C. *et al.* High-Resolution Global Maps of 21st-Century Forest Cover Change. *Science* **342**, 850–853 (2013).
